# Single-sensor system for spatially resolved, continuous, and multiparametric optical mapping of cardiac tissue

**DOI:** 10.1016/j.hrthm.2011.03.061

**Published:** 2011-09

**Authors:** Peter Lee, Christian Bollensdorff, T. Alexander Quinn, Joseph P. Wuskell, Leslie M. Loew, Peter Kohl

**Affiliations:** ⁎ Cardiac Mechano-Electric Feedback Lab, Department of Physiology, Anatomy and Genetics, University of Oxford, Oxford, United Kingdom; † Life Sciences Interface Doctoral Training Centre, University of Oxford, Oxford, United Kingdom; ‡ Cardiac Biophysics and Systems Biology Group, National Heart and Lung Institute, Imperial College, London, United Kingdom; § Richard D. Berlin Center for Cell Analysis and Modeling, University of Connecticut Health Center, Farmington, Connecticut

**Keywords:** Arrhythmia, Electrophysiology, Fluorescence, Mechano-electric coupling, Optical mapping, AP, action potential, [Ca^2+^]_i_, intracellular free calcium, CaT, Ca^2+^ transient, EMCCD, electron-multiplied charge-coupled device, LED, light-emitting diode, UV, ultraviolet, V_m_, membrane potential

## Abstract

**Background:**

Simultaneous optical mapping of multiple electrophysiologically relevant parameters in living myocardium is desirable for integrative exploration of mechanisms underlying heart rhythm generation under normal and pathophysiologic conditions. Current multiparametric methods are technically challenging, usually involving multiple sensors and moving parts, which contributes to high logistic and economic thresholds that prevent easy application of the technique.

**Objective:**

The purpose of this study was to develop a simple, affordable, and effective method for spatially resolved, continuous, simultaneous, and multiparametric optical mapping of the heart, using a single camera.

**Methods:**

We present a new method to simultaneously monitor multiple parameters using inexpensive off-the-shelf electronic components and no moving parts. The system comprises a single camera, commercially available optical filters, and light-emitting diodes (LEDs), integrated via microcontroller-based electronics for frame-accurate illumination of the tissue. For proof of principle, we illustrate measurement of four parameters, suitable for ratiometric mapping of membrane potential (di-4-ANBDQPQ) and intracellular free calcium (fura-2), in an isolated Langendorff-perfused rat heart during sinus rhythm and ectopy, induced by local electrical or mechanical stimulation.

**Results:**

The pilot application demonstrates suitability of this imaging approach for heart rhythm research in the isolated heart. In addition, locally induced excitation, whether stimulated electrically or mechanically, gives rise to similar ventricular propagation patterns.

**Conclusion:**

Combining an affordable camera with suitable optical filters and microprocessor-controlled LEDs, single-sensor multiparametric optical mapping can be practically implemented in a simple yet powerful configuration and applied to heart rhythm research. The moderate system complexity and component cost is destined to lower the threshold to broader application of functional imaging and to ease implementation of more complex optical mapping approaches, such as multiparametric panoramic imaging. A proof-of-principle application confirmed that although electrically and mechanically induced excitation occur by different mechanisms, their electrophysiologic consequences downstream from the point of activation are not dissimilar.

## Introduction

Optical imaging has had a profound impact on understanding excitable tissue physiology. In particular, optical mapping of electrophysiologic parameter-sensitive dyes has contributed to a better understanding of action potential (AP) generation and conduction dynamics in multicellular preparations so that fluorescence imaging has become a gold standard for functional research (for review see Efimov et al^1^). However, the technical complexity of measuring more than one parameter and the associated cost have impeded broad uptake of multiparametric imaging as a standard research tool.

Modern fluorescent probes can selectively report membrane potential (V_m_), pH, and ion concentrations such as intracellular free calcium ([Ca^2+^]_i_), and it is possible to combine certain probes in one preparation.^2^ For heart rhythm research, combined V_m_ and [Ca^2+^]_i_ monitoring is arguably of particular relevance, as these parameters underlie electromechanical integration. Normal excitation–contraction coupling involves V_m_ depolarization, transsarcolemmal Ca^2+^ influx, bulk Ca^2+^ release from the sarcoplasmic reticulum, resulting in increased [Ca^2+^]_i_ forming a Ca^2+^ transient (CaT). Via interaction with troponin-C, CaT enables cross-bridge interactions for myocardial force generation/shortening. Excitation–contraction coupling–related interactions between V_m_ and [Ca^2+^]_i_ are complemented by mechano-electric feedback,^3^ where the mechanical environment affects Ca^2+^ buffering^4^ and fluxes across sarcolemmal^5^ and intracellular membranes in cardiomyocytes^6^ and nonmyocytes,^7^ with consequences for electrical behavior, including induction of premature ventricular beats.^8^

Thus, V_m_ and [Ca^2+^]_i_ interactions are dynamic and complex. Underlying mechanisms are modulated by pathologies such as ischemia, with relevance for cardiac mechanical and electrical performance, including arrhythmogenesis.^9–11^ Therefore, simultaneous V_m_ and CaT mapping in one and the same sample is desirable for probing spatiotemporal relationships of these key (patho-)physiologically relevant parameters.

Simultaneous V_m_ and CaT measurements have been achieved in myocardial preparations, including whole heart.^12–15^ Utilizing appropriate probe/filter combinations, V_m_ and CaT have been imaged separately, using two cameras. Although multiparametric optical mapping is still relatively uncommon, the insight gleaned from such simultaneous measurements, particularly at high spatiotemporal resolution, is extremely promising, and wider application in cardiac research appears desirable.^16^

Technical limitations of currently available optical mapping approaches include uneven dye loading and/or illumination, photobleaching, and imaging artifacts induced by contraction (so-called “motion artifacts”). Emission ratiometry with V_m_-sensitive dyes, such as di-4-ANEPPS,^17^ or nonratiometric Rh237 combined with calcium probes such as fluo-4 or Oregon-green BAPTA-1,^18^ have been used to reduce or partially correct these effects. Second-generation ratiometric calcium probes (fura-2, indo-1) have been used to characterize absolute [Ca^2+^]_i_ levels,^19,20^ although [Ca^2+^]_i_ calibration and motion tracking remain challenging in multicellular preparations (and are outside the scope of the present methods development; for review see Entcheva and Bien^21^).

Ratiometric dyes display a spectral shift (e.g., on Ca^2+^ binding),^22^ which changes with exposure to different excitation wavelengths on opposite sides of the “emission isosbestic point” (the excitation wavelength at which a change in reported parameters produces no change in emission). On either side of this isosbestic point, alterations in emission intensity are of opposite polarity. The ratio of these two emission intensities is independent of fluorescence intensity so that some of the above-stated limitations (e.g., uneven dye loading) can be addressed. Inherent to multiwavelength mapping is that light of different wavelengths penetrates tissue to varying degrees, which may affect recorded light intensities. This need not be a restriction because it can be used to explore depth-weighted behavior.^23,24^

To exploit the full potential of ratiometric techniques for multiparametric optical mapping, simple, robust, and scalable approaches to multiwavelength imaging are needed. Thus far, dedicated cameras have generally been used to collect light at different wavelengths. This makes multiparametric mapping challenging. Difficulties include detector alignment (i.e., matching camera pixels), intensity loss due to extended light paths and additional components (e.g., beam splitters), and positioning in a restricted space. Even if one divided a single sensor into separate areas, dedicated to collection of separate wavelengths, optical alignment remains challenging if more than two parameters are involved.

That said, modern camera frame rates permit multicolor imaging using a single detector if combined with suitable excitation timing and emission filtering. In this context, multiband emission filtering^25^ offers exciting possibilities for cardiac optical mapping.

Traditional wide-field imaging light sources include xenon, halogen, or mercury lamps in combination with mechanical shutters and filter wheels to switch excitation wavelengths. This imposes limitations on wavelength switching and scalability. In addition, altering individual wavelength intensity in broad-band sources is nontrivial, requiring neutral density filters. These limitations can be overcome by light-emitting diodes (LEDs), which provide stable, flexible, and economical alternatives to previous lighting methods. For example, unlike traditional sources, LED light intensities can be modulated exceedingly fast (microsecond domain; e.g., Figure 3C). Powerful LED chips are available now from ultraviolet (UV) to infrared, making them attractive for multiwavelength imaging, such as of V_m_ and CaT in myocardium, where deep UV-LED illumination has unexplored potential.^26^

Using standard mapping approaches, the study of normal and pathophysiologically disturbed interactions of V_m_ and [Ca^2+^]_i_ would require at least two, potentially four, cameras for ratiometric measurements of single-excitation/dual-emission dyes. Here, we present a method to simultaneously measure V_m_ and CaT using two excitation wavelengths for each parameter and only a single camera. This is combined with readily available filters and LEDs, integrated by custom-made microcontroller-based electronics, using off-the-shelf components. We used the novel V_m_-sensitive di-4-ANBDQPQ^27^ and the calcium dye fura-2 to demonstrate the applicability of this method to multiparametric studies in Langendorff-perfused rat hearts. As proof of principle, we illustrate that ectopic excitation following local electrical or mechanical stimulation gives rise to similar propagation patterns.

## Materials and methods

For expanded methods including circuit diagrams, parts listings, and software description, see the Online Supplemental Data.

## Isolated heart preparation

Hearts were isolated from female Wistar rats (weight 250–350 g) after cervical dislocation in accordance with Schedule 1 of the UK Home Office Animals (Scientific Procedures) Act of 1987. For proof-of-principle multiparametric optical mapping, contractile motion was minimized by the excitation–contraction coupling uncoupler blebbistatin (10 μmol × L^–1^).^28^ Fluorescent dyes were applied either by bolus injection into the aortic cannula (di-4-ANBDQPQ: 20 μL of 27.3 mmol × L^–1^ in ethanol, added over 5 minutes to 25 mL of perfusate) or by recirculation (fura-2: 10 μmol × L^–1^ over 30 minutes).

## Instrumentation

**Figure 1:**
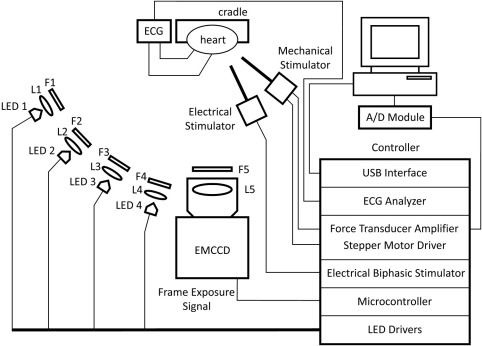
Simplified scheme of the imaging setup for spatially resolved single-sensor multiparametric optical mapping, combined with local electrical or mechanical stimulation (see text for details). A/D module = analog-to-digital converter; controller = microcontroller-based interface; ECG = ECG recorder with spring-loaded Ag/AgCl pellet electrodes; EMCCD = fluorescence imaging camera; F1–4 = band-pass excitation filters (see Figure 2); F5 = multiband emission filter (see Figure 2); L1–4 = collimating lenses; L5 = camera lens; LED1–4 = LED light sources (two each).

**Figure 2:**
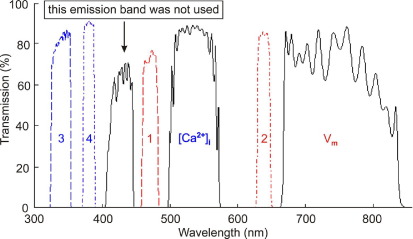
Transmission spectra of the four excitation filters *(interrupted lines)* and the single multiband emission filter (*solid lines;* see text for details). Numbers 1–4 relate to excitation filters 1–4 described in Methods. V_m_ and [Ca^2+^]_i_ indicate emission-filter wavelength bands used to detect di-4-ANBDQPQ and fura-2 signals (short wavelength band of emission filter was not used). For information on dye emission spectra, see Matiukas et al^27^ (di-4-ANBDQPQ) and Grynkiewicz et al^22^ (fura-2). Curves showing filter characteristics compiled from Chroma Technology and Semrock catalogue data.

A schematic setup is shown in Figure 1. LED-based illumination (four different wavelengths) was collimated (planoconvex spherical lenses; Thorlabs, Newton, NJ, USA), passed through excitation filters (Figure 2), and focused on the heart's surface. Although only one light source is shown per wavelength, two equal LEDs each were used for more even illumination. Fluorescence emission from dye-loaded myocardium was passed through one multiband emission filter (Figure 2) and collected by a fast camera suitable lens (f/#0.95; DO-2595; Navitar, Rochester, NY, USA). Fluorescence was recorded with an electron-multiplied charge-coupled device (EMCCD; Cascade-128+: 128 × 128, 24-μm-square pixels, 16 bit; Photometrics, Tucson, AZ, USA; EM gain was turned off, so the camera was used in CCD mode).

A custom-built microcontroller-based interface (1) synchronized LED switching with the EMCCD camera's frame exposure signal and (2) controlled timing of local electrical/mechanical stimulation relative to the ECG. LEDs were driven with a fast high-power circuit that enables swift illumination power adjustment and switching (in the kHz range). Circuitry for electrical (biphasic) and mechanical stimulation was custom built and linked to a concentric-bipolar stimulation electrode (Lohmann Research Equipment, Castrop-Rauxel, Germany) and a stepper motor (Lin Engineering, Morgan Hill, CA, USA) coupled to a single-axis mechanical manipulator (Edmund Optics, York, United Kingdom), respectively. To enable force measurement, the mechanical stimulator probe was mounted to a force transducer (UF1; LCM Systems, Newport, United Kingdom), whose output was sampled with an analog-to-digital converter (MP150; BIOPAC Systems, Goleta, CA, USA). To time electrical/mechanical stimulus application, the ECG was recorded (ECG100C; BIOPAC Systems) and differentiated using analog circuitry to detect peak slope of QR upstroke. All stimuli were applied at specified times relative to this peak.

An eight-processor microcontroller (Propeller-Chip; Parallax, Rocklin, CA, USA) was used to control/coordinate all major components. Control software for time-critical tasks was written in the microcontroller's assembly language to ensure a time resolution of 50 ns. Communication with a desktop computer occurred via USB interface (UM245R; Future Technology Devices, Glasgow, United Kingdom). Custom software written in MATLAB (The MathWorks, Natick, MA, USA) was used to design experimental protocols, communicate with the microcontroller, and perform optical image processing.

Our method can be adapted to camera systems other than the model used here, as long as they provide a frame exposure signal. LED sources, along with collimating lenses, band-pass filters, and microcontroller-based interface, represent an inexpensive (<$1,500) and versatile add-on to existing systems. All components for the microcontroller-based interface (Figure 1) were acquired from major components distributors.

## Single-sensor multiparametric optical mapping

For single-sensor concurrent ratiometric V_m_ and CaT mapping, we selected dual-excitation/single-emission ratiometric dyes. The excitation sources for di-4-ANBDQPQ were (1) LED-CBT-90-B (peak output 53 W, peak wavelength 460 nm; Luminus Devices, Billerica, MA, USA) with excitation filter D470/20x (Chroma Technology, Bellows Falls, VT, USA) and (2) LED-CBT-90-R (peak output 32 W, peak wavelength 628 nm; Luminus Devices) with excitation filter D640/20x (Chroma Technology), which we refer to as excitation sources Ex1 and Ex2, respectively (see Figure 2 for filter spectra). The excitation sources for fura-2 were (1) LED-UVMAX325-HL-15 (peak output 15 mW, peak wavelength 330 nm, Roithner LaserTechnik, Vienna, Austria) with excitation filter FF01-340/26 (Semrock, Rochester, NY, USA) and (2) LED-NCSU034A (peak output 400 mW, peak wavelength 385 nm; Nichia, Tokushima, Japan) with excitation filter FF01-380/14 (Semrock), which we refer to as excitation sources Ex3 and Ex4, respectively. Ex1 and Ex2 produced fluorescence emission in the third emission filter band (V_m_); Ex3 and Ex4 produced fluorescence emission in the second emission filter band ([Ca^2+^]_i_; Figure 2).

During any EMCCD frame exposure period, the heart was illuminated with only one excitation wavelength. Demarcation between excitation and emission wavelengths allowed avoidance of cross-talk between V_m_ and CaT signals so that emitted fluorescence represents one of two ratiometric V_m_ emissions or one of two ratiometric [Ca^2+^]_i_ emissions, as confirmed by single-dye control studies.

## Results

**Figure 3:**
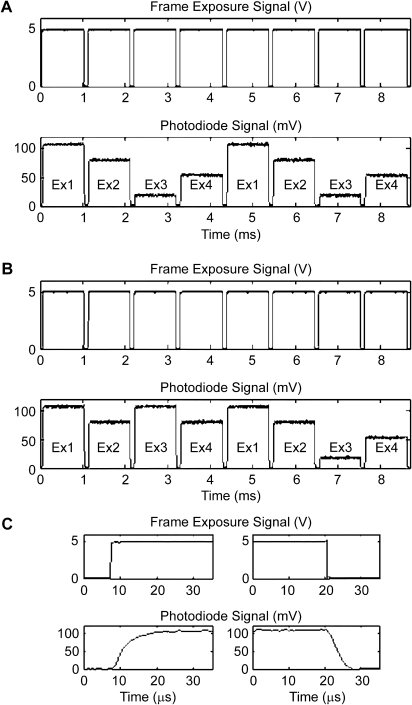
Oscilloscope traces of camera frame exposure (*top traces;* 5 V = on) and photodiode signals, recorded from four different LED excitation sources (*bottom traces*) that were triggered by the camera frame exposure signal. Ex1–4 correspond to excitation sources 1–4 (see Figure 2). **A:** Excitation sources were turned on and off sequentially and in a fixed sequential pattern. **B:** Excitation sources 1 and 2 were activated 3× more often than sources 3 and 4. **C:** Expanded recording of photodiode signal collected from Ex1, illustrating rise and fall times of LED illumination (10^–5^-second range).

To characterize the coordination between frame exposure and LED excitation, we built a simple light detector based on a high-speed photodiode (S5971, Hamamatsu Photonics, Shizuoka, Japan). The distance between excitation sources (output power was set to values typically used in experiments) and photodiode was chosen such that individual LED sources could be identified based solely on the amplitude of the recorded photodiode output. Figure 3 shows the camera frame exposure signal (generated by the EMCCD, acting as “master” timer to control LED switching) and LED illumination captured by the photodiode. Figure 3A illustrates that excitation sources were turned on/off in a nonoverlapping sequential manner. In the given example (EMCCD frame rate 930 Hz, 64 × 64 pixel frame), this means that the “sampling rate per wavelength” is 232.5 Hz, whereas the “sampling rate per parameter” is 465 Hz.^1^ A different excitation sequence is shown in Figure 3B, where sources 1 and 2 were activated 3× more often than sources 3 and 4. The latter sequence represents a pattern optimized to capture more of the faster dynamics of V_m_ (here sampled at 697.5 Hz) compared to CaT (here at 232.5 Hz).

**Figure 4:**
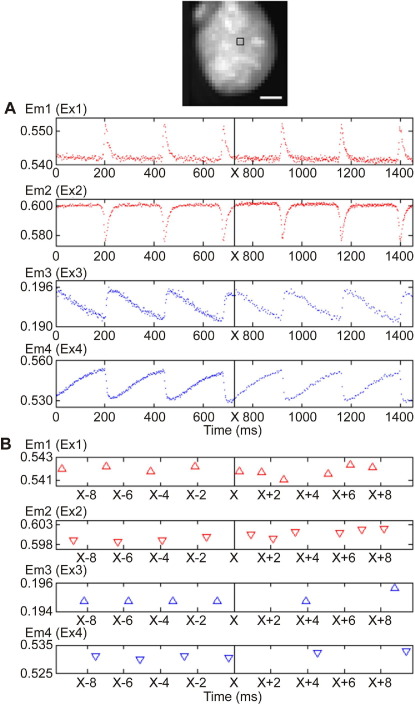
Multiparametric optical mapping of rat left ventricle with “on-the-fly” change in excitation sequence (unfiltered data; points plotted are obtained from the 2 × 2 pixel area outlined by the *black square, top panel*, shown without interpolation). Em1–4 correspond to emission fluorescence during exposure to excitation sources Ex1–4, respectively. All four excitation sources are initially turned on and off sequentially. At time “X,” this sequence is changed to one where sources 1 and 2 are activated 3× more often than sources 3 and 4. **A:** Optical mapping data for V_m_*(top two traces; red)* and CaT *(bottom two traces; blue).***B:** Expanded view, in time and amplitude, of the transition in excitation pattern. Scale bar = 5 mm.

A recording of V_m_ and CaT during “on-the-fly” changes in excitation sequence (which would be challenging using traditional mechanical switching approaches) from the initial even sequential to a V_m_-weighted pattern is illustrated in Figure 4, demonstrating the flexibility afforded by LED excitation sources. Here, the EMCCD frame rate was 1,656 Hz (32 × 32 pixel frame), initially sampling both V_m_ and CaT at 828 Hz, then switching to 1,242 Hz for V_m_ and 414 Hz for CaT. More complex switching patterns (e.g., ECG-driven preference periods for a parameter) are possible (e.g., to optimize sampling rates to periods of fastest change in any observed parameter). Within the rate requirements typically associated with cardiac electrophysiology research, this is not limited by LED switching speeds. Figure 3C shows the fast rise and fall times of LED emission (with increased temporal resolution, here for Ex1). Rise times for all LEDs were 8–12 μs and fall times never exceeded 10 μs; both are significantly less than the fixed 100-μs time gap between individual EMCCD frame exposures, allowing frame-accurate LED switching.

To establish whether the presence of multiple dyes affects observed parameters, control experiments compared emission during single-dye loading with dual-dye data in the same heart (n = 3 for each loading sequence). In these experiments, the EMCCD camera was set to a 64 × 64 pixel frame (illumination sequence shown in Figure 3A).

**Figure 5:**
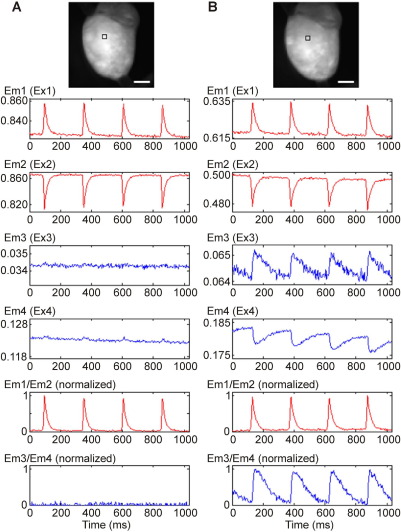
Multiparametric optical mapping of rat right ventricle (original traces, unfiltered, obtained from tissue within 4 × 4 pixel square in *top panel*). Em1–4 correspond to emitted fluorescence during exposure to excitation sources Ex1–4, respectively. **A:** In the presence of the V_m_-sensitive di-4-ANBDQPQ only, no significant fluorescence signal is detected when illuminated by excitation sources 3 and 4 (excitation sources for fura-2). **B:** Simultaneous ratiometric V_m_ and ratiometric [Ca^2+^]_i_ optical mapping after additional loading of the same heart with fura-2. EM1/EM2 and EM3/EM4 show normalized V_m_ and CaT signals after calculation of corresponding source signal ratios (EM3/EM4 signals in **A** are normalized with respect to corresponding signal-amplitudes in **B**). Scale bar = 5 mm. Note two to 20-fold difference in Y-axis scaling for Em3 and Em4, compared to EM1 and EM2.

**Figure 6:**
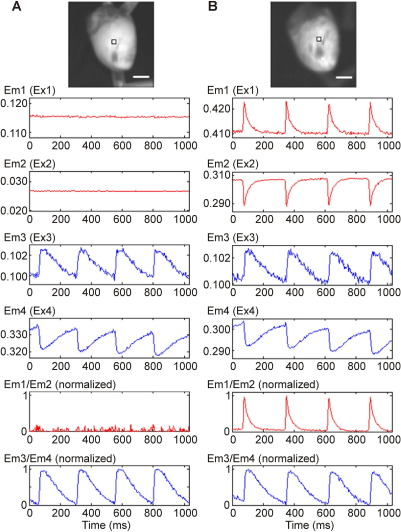
Multiparametric optical mapping of rat left ventricle (original signal traces, unfiltered, obtained from tissue within 4 × 4 pixel square outlined in *top panel*). Em1–4 correspond to emitted fluorescence during exposure to excitation sources Ex1–4, respectively. **A:** In the presence of the [Ca^2+^]_i_ dye fura-2 only, no significant fluorescence signal is detected when illuminated by sources 1 and 2 (excitation sources for di-4-ANBDQPQ). **B:** Simultaneous ratiometric V_m_ and ratiometric [Ca^2+^]_i_ optical mapping after additional loading with di-4-ANBDQPQ. EM1/EM2 and EM3/EM4 show normalized V_m_ and CaT signals after calculation of corresponding source signal ratios (EM1/EM2 signals in **A** are normalized with respect to corresponding signal amplitudes in **B**). Scale bar = 5 mm. Note up to 10-fold difference in Y-axis scaling for Em3 and Em4, compared to EM1 and Em2.

In the first set of control experiments (Figure 5), hearts were loaded initially with di-4-ANBDQPQ only, and emission was collected during exposure to all four excitation wavelengths. Only excitation Ex1 and Ex2 yielded V_m_ signals (Figure 5A; recordings are unfiltered “raw” data). After additionally loading the same hearts with fura-2 (Figure 5B), no significant change in V_m_ signals was found, even though the loading procedure for the Ca^2+^ dye required a significant amount of time (recordings in Figures 5A and 5B were obtained more than 1 hour apart). Signal-to-noise ratios were assessed by dividing the power of the signal (defined as the amplitude of change in fluorescence) by the power of the noise, yielding the following values (rounded to nearest whole number): Em1 = 360, Em2 = 170, Em3 = 8, Em4 = 155, Em1/Em2 = 506, Em3/Em4 = 88. In the second set of control experiments (Figure 6), hearts were initially loaded with fura-2 only and cycled through all four excitation wavelengths. Only Ex3 and Ex4 yielded CaT signals (Figure 6A). After addition of di-4-ANBDQPQ to the same heart (Figure 6B), no significant changes in CaT were observed (recordings in Figures 6A and B are more than 1 hour apart, illustrating satisfactory signal stability). Corresponding signal-to-noise ratio values were Em1 = 200, Em2 = 250, Em3 = 54, Em4 = 218, Em1/Em2 = 337, Em3/Em4 = 222.

These tests confirm the viability of the proposed multiparametric optical mapping method (see Online Supplement Figure I for representative time series of ratiometric V_m_ and Ca^2+^ maps during spontaneous sinus node activity). However, loading hearts with fura-2 before di-4-ANBDQPQ yielded better overall CaT signals, so this sequence was utilized henceforth.

**Figure 7:**
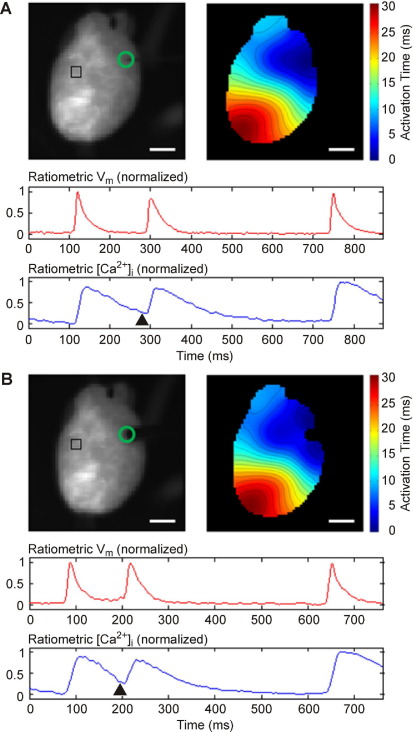
Ratiometric V_m_ and CaT signals (obtained from tissue within 8 × 8 pixel region of *top left panels* in **A** and **B**) were smoothed with a moving-average box filter (using side lengths of 3 and 13 pixels for V_m_ and CaT, respectively) and collected during sinus activation, interrupted by ectopic excitation triggered either (**A**) electrically (here 170 ms after peak QR upstroke slope) or (**B**) mechanically (here 140 ms after peak QR upstroke slope). *Arrowheads* below CaT curve indicate stimulus timing. Activation maps (*top right panels* of **A** and **B**) indicate spatial distribution of activation timings, derived from maximum voltage upstroke velocity, with isochrones 2 ms apart. Electrical and mechanical stimuli were applied near the right ventricular base (*circle* in *top left panels* of **A** and **B**). Note postextrasystolic potentiation of CaT following pause after the ectopic beat, typical for rat ventricular myocardium. Scale bar = 5 mm.

A proof-of-principle application explored effects of local electrical and mechanical stimulation, applied during diastole to the same right ventricular epicardial surface area (free wall, near base; n = 4), to induce ectopic excitation. Figure 7 shows simultaneous ratiometric recordings of V_m_ and CaT during sinus node activity, interrupted by either electrically (Figure 7A) or mechanically (Figure 7B) induced ectopy. The electrical stimulus was a bipolar pulse (amplitude ∼4 V, duration 3 ms). The mechanical stimulus involved application of forces (up to 150 mN and 100 ms) to a contact area of 1.65 mm^2^. Amplitudes of mechanical and electrical stimuli were set at 50% above threshold for ectopic excitation in any given preparation.

**Figure 8:**
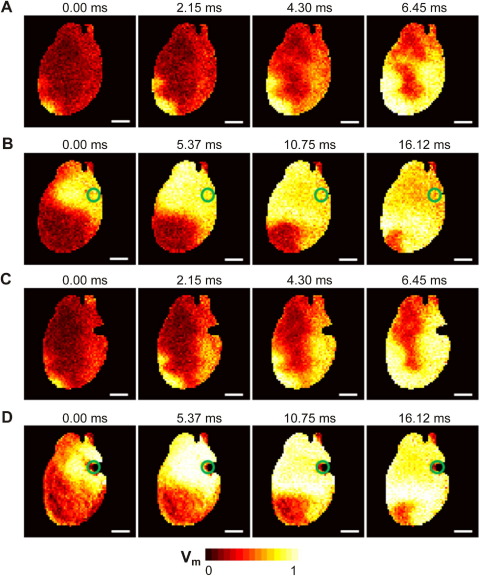
Optical mapping of electrically and mechanically induced ectopic excitation in rat heart (color coding represents normalized ratiometric V_m_ signals; see color bar). *Green circles* indicate location of focal stimulus site. **A:** Sinus excitation just prior to (B). **B:** Electrically induced ectopic excitation. **C:** Sinus excitation just prior to (D). **D:** Mechanically induced ectopic excitation (apparently brighter signal intensity, compared to electrical activation, is caused by slight impact-induced motion). Scale bar = 5 mm.

No significant differences were observed between conduction patterns of electrically or mechanically induced ectopic excitation (Figure 7), including apparent epicardial conduction velocity (0.658 ± 0.076 m × s^–1^) and electromechanical coupling time, assessed as the delay between the peaks of V_m_ and CaT (20.2 ± 2.59 ms; see Online Supplement Figures II and III, and corresponding analyses). Figure 8 shows example optical maps of sinus node activation (Figures 8A and 8C) and of electrically or mechanically induced ectopy (Figures 8B and 8D, respectively).

## Discussion

Taking advantage of technology developments in high-speed cameras, multiband optical filtering, solid-state illumination, and control electronics, we developed a simple, versatile, and scalable system to concurrently measure multiple fluorescent parameters, here applied to V_m_ and CaT in isolated Langendorff-perfused hearts, using a single camera. Key benefits of this approach include ease of implementation, compactness of instrumentation, simplicity of control, lack of moving parts, versatility of application, quality of data, compatibility with existing optical mapping setups, and low cost.

As investigations into electromechanical cross-talk in the heart continue to uncover new insight,^29^ we developed a mapping-compatible method to apply to the same location either electrical or mechanical stimuli. For proof of principle, we compared electrically and mechanically induced AP conduction and found no significant differences in propagation speed and patterns, or V_m_ to CaT peak delay. Therefore, this study confirms in whole heart previous observations in cultured myocytes monolayers^30^ and validates the similarity of downstream effects of ectopically triggered excitation, regardless of stimulus nature, as had been assumed in previous conceptual and computational modelling.^31,32^

On the imaging side, key methodologic advances of this work include implementation of a simple, versatile, and efficient approach to multiparametric mapping, using a single detector. Although powerful LEDs with emission in the visible light spectrum have been available for several years, LEDs with sufficiently intense UV emission (down to 300 nm) have emerged only recently. Their availability opens up novel opportunities due to the array of dyes and compounds that respond to UV illumination.

On the electronics instrumentation side, the integration of microcontrollers into cardiovascular research extends experimental possibilities by improving temporal resolution and expanding the range of feasible control procedures. The recently developed microcontroller used here contains eight parallel processors and is ideal for laboratory instrumentation and automation.

V_m_ dyes have vastly improved in terms of ease of loading, voltage sensitivity, and internalization stability. The near-infrared di-4-ANBDQPQ not only provides improved V_m_ signals for short-term and long-term measurements but also enables new dye combinations due to its long wavelength excitation/emission spectra, as illustrated by concurrent V_m_ and CaT monitoring using di-4-ANBDQPQ and fura-2.

Thus, the described method benefits from several recent consumables and device developments and, by combining them in a simple, compact, and effective layout, offers a significant reduction in technical complexity while maintaining sufficient signal quality to conduct multiwavelength investigations. The set of four wavelengths chosen here is simply an example, as fewer, more, or different wavelengths may be monitored, provided excitation and emission spectra can be separated. The use of deep-UV LEDs for cardiac research, multiband emission filters, and spatially resolved ratiometric fura-2 mapping in the whole-heart (i.e., using a camera rather than single-element sensors or small arrays) are additional novel aspects of the method.

At the same time, our method has certain real or perceived limitations. Single-sensor multiparametric optical mapping relies on the lack of cross-talk between dyes, which restricts possible parameter combinations. However, this limitation applies to all multimodal imaging techniques. In addition, the camera must be fast enough to share frame exposures among multiple excitation/emission combinations because it relies on collecting only one relevant emission wavelength during any frame exposure. This may require sampling at submaximal spatial resolutions (largely, we used 64 × 64 pixels, even though the chip contains 128 × 128). Given the rate of improvements in spatiotemporal camera resolution and sensitivity, the impact of this limitation will become increasingly less problematic. Furthermore, tissue mapping can be affected by differential attenuation of excitation or emission light at different wavelengths. Again, this is not specific to the approach presented here.^33^ Application to preparations of reduced thickness (e.g., tissue slices)^34^ may hold interesting potential because these experimental models are more representative of *in situ* structure and function and are less photosensitive than monolayers or cells. Finally, a perceived limitation is that individual wavelengths are collected at subsequent points in time (here ∼1 ms nonoverlapping frame sequences), which may be regarded as nonsimultaneous parameter monitoring. However, interpolation between datapoints allows creation of a continuous curve representative of underlying physiologic behavior, as long as sampling rates are fast enough to capture fast-/short-lived changes (a prerequisite common to all experimental data gathering). For all intents and purposes, therefore, the present technique allows concurrent characterization of multiparametric behavior using only a single sensor.

## Conclusion

We present a simple, compact, effective, and affordable method for spatially resolved, continuous, concurrent, and multiparametric optical mapping using a single camera. For proof of principle, we monitored V_m_ and CaT in isolated Langendorff-perfused rat hearts and observed that excitation wave dynamics downstream of electrically and mechanically induced ectopic foci are similar, regardless of the stimulus modality. With increasing interest in, and demand for, even more advanced imaging approaches, from motion tracking to three-dimensional panoramic mapping,^35^ simplified multiparametric systems such as this may allow implementation of study designs that presently are outside the realm of experimental and financial viability.

## Footnotes

1 The two emission datasets that characterize one and the same physical parameter were taken with a phase shift of half a sampling cycle. Because emission at both excitation wavelengths would be affected by any sudden change in underlying physical parameters, this approach justifies interpolation between raw datapoints obtained with one excitation wavelength, as long as there are no erratic changes in emission intensity obtained with the other wavelength. This permits calculation of one ratiometric data value at every timepoint for which one emission datapoint is obtained, i.e., here at 232.5 Hz + 232.5 Hz = 465 Hz.
