# Supplementary material for: Single-sensor system for spatially resolved, continuous, and multiparametric optical mapping of cardiac tissue
Source: Heart Rhythm. 2011 Sep;8(9):1482–91. doi: 10.1016/j.hrthm.2011.03.061 (PMC3167353; doi:10.1016/j.hrthm.2011.03.061)
Supplement: Supplemental Data [file mmc1.doc]

**Supplemental Material for Lee *et al*. “Single-Sensor System for Spatially-Resolved, Continuous and Multi-Parametric Optical Mapping of Cardiac Tissue”**

**Detailed Methods**

**Tissue Preparation**

Hearts were isolated from female Wistar rats (n=13), weighing 250–350 g, after cervical dislocation, in accordance with Schedule 1 of the UK Home Office Animals (Scientific Procedures) Act of 1987, and swiftly connected to a Langendorff perfusion setup. Hearts were positioned in a lab-film cradle shaped to optimally suite the individual sample, and perfused at a constant rate of 5 mL x min^-1^ with normal Krebs-Henseleit solution (containing, in mmol × L^-1^: NaCl 123, CaCl_2_ 1.8, KCl 4, MgCl_2_ 1.2, NaH_2_PO_4_ 1.4, NaHCO_3_ 24, Glucose 10; bubbled with 95% O_2_/5% CO_2_; pH 7.4; osmolarity 300 ± 3 mOsm; excitation-contraction uncoupled with blebbistatin (Sigma-Aldrich) 10 µmol × L^-1^). All experiments were conducted at 36±1 ^o^C.

Fluorescent dyes were injected into the aortic cannula for coronary perfusion. To image Ca^2+^ transients (CaT), hearts were stained by re-circulating perfusion with 50 mL of 10 µmol x L^-1^ fura-2AM (Sigma-Aldrich) for 30 minutes.^1^ To image V_m_, the myocardium was stained by delivering, without recirculation, a 20 µL bolus of 27.3 mmol × L^-1^ (in pure ethanol) di-4-ANBDQPQ (Richard D. Berlin Center for Cell Analysis and Modeling, University of Connecticut Health Center), applied over 5 minutes (i.e. diluted in 25 mL perfusate).^2^ To load di-4-ANBDQPQ, Pluronic F-127 (Sigma-Aldrich) was added to the bolus, to a final concentration of 0.2 - 0.5%.

**Supplemental Figures and Figure Narratives**


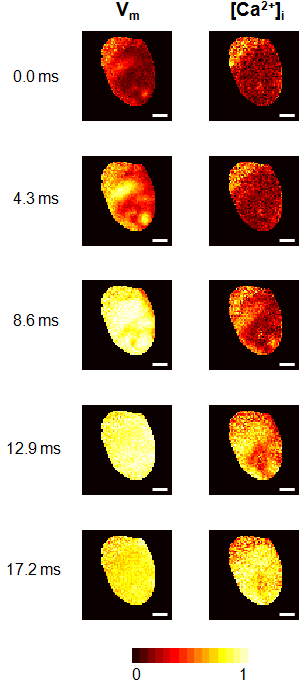


**Online Figure I** Optical mapping of normalized ratiometric V_m_ and CaT ([Ca^2+^]_i_) signals; colour coding illustrates relative amplitudes (see colour bar at the bottom). Note the excitation-contraction coupling delay between the V_m_ peak and CaT peak signals. Scale bar: 5 mm.


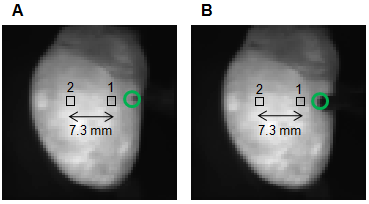


**Online Figure II** Comparing electrically and mechanically induced ectopic excitation, triggered near the base of the right ventricular free wall in isolated Langendorff-perfused rat heart: equatorial direction. **A:** Electrically induced ectopic beat. **B:** Mechanically induced ectopic beat. Green circle: location of stimulus application. 4x4 pixel squares represent tissue regions analyzed below.

**Corresponding Analysis:**

Delay1E: V_m_-peak to CaT-peak delay for the sinus beat two cycles before the electrically induced ectopic beat (black square 2)

24.2 ± 1.49 ms (n = 8)

Delay1M: V_m_-peak to CaT-peak delay for the sinus beat two cycles before the mechanically induced ectopic beat (black square 2)

23.8 ± 1.67 ms (n = 8)

Delay2E: V_m_-peak to CaT-peak delay for the electrically induced ectopic beat (black square 2)

18.2 ± 1.03 ms (n = 8)

Delay2M: V_m_-peak to CaT-peak delay for the mechanically induced ectopic beat (black square 2)

18.2 ± 2.55 ms (n = 8)

Delay3E: Time delay between V_m_-peaks in regions outlined by black squares 1 and 2 for the electrically induced ectopic beat

11.0 ± 0.756 ms (n = 8)

(apparent speed of propagation from black square 1 to 2: ~ 0.66 mm × ms^-1^)

Delay3M: Time delay between V_m_-peaks in regions outlined by black squares 1 and 2 for the mechanically induced ectopic beat

11.4 ± 0.916 ms (n = 8)

(apparent speed of propagation from black square 1 to 2: ~ 0.64 mm × ms^-1^)

Delay1E vs. Delay1M:

Welch’s t-test: t = 0.506 < 2.145 (no significant difference between the two delays at P = 0.05)

Delay2E vs. Delay2M:

Welch’s t-test: t = 0.0 < 2.262 (no significant difference between the two delays at P = 0.05)

Delay3E vs. Delay3M:

Welch’s t-test: t = 0.953 < 2.145 (no significant difference between the two delays at P = 0.05)


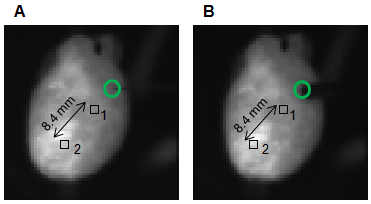


**Online Figure III** Comparing electrically and mechanically induced ectopic excitation, triggered near the base of the right ventricular wall in isolated Langendorff-perfused rat heart: axial direction. **A:** Electrically induced ectopic beat. **B:** Mechanically induced ectopic beat. Green circle: location of stimulus application. 4x4 pixel squares represent tissue regions analyzed below.

**Corresponding Analysis:**

Delay1E: V_m_ peak to CaT peak delay for the sinus beat two cycles before the electrically induced ectopic beat (black square 2)

25.2 ± 1.83 ms (n = 8)

Delay1M: V_m_ peak to CaT peak delay for the sinus beat two cycles before the mechanically induced ectopic beat (black square 2)

24.6 ± 1.99 ms (n = 8)

Delay2E: V_m_ peak to CaT peak delay for the electrically induced ectopic beat (black square 2)

22.0 ± 1.41 ms (n = 8)

Delay2M: V_m_ peak to CaT peak delay for the mechanically induced ectopic beat (black square 2)

22.5 ± 1.31 ms (n = 8)

Delay3E: Time delay between V_m_-peaks in regions outlined by black squares 1 and 2 for the electrically induced ectopic beat

13.1 ± 0.834 ms (n = 8)

(apparent speed of propagation from black square 1 to 2: ~ 0.64 mm × ms^-1^)

Delay3M: Time delay between V_m_-peaks in regions outlined by black squares 1 and 2 for the mechanically induced ectopic beat

12.9 ± 1.64 ms (n = 8)

(apparent speed of propagation from black square 1 to 2: ~ 0.65 mm × ms^-1^)

Delay1E vs. Delay1M:

Welch’s t-test: t = 0.626 < 2.145 (no significant difference between the two delays at P = 0.05)

Delay2E vs. Delay2M:

Welch’s t-test: t = 0.734 < 2.145 (no significant difference between the two delays at P = 0.05)

Delay3E vs. Delay3M:

Welch’s t-test: t = 0.307 < 2.228 (no significant difference between the two delays at P = 0.05)

As an illustration, assessment of the V_m_-peak to CaT-peak delay shows no differences for electrically and mechanically induced ectopic excitation.

All analysis was conducted on the ratiometric data, as this is less sensitive to differences in dye loading, illumination, or photo-bleaching. This data was also less sensitive to minor signal bleed-through, as observed, for example, in the form of small ‘blips’ in Em4 of Fig.4A. This cross-talk was minor enough to permit analysis. As this was due to limitations in pre-existing multi-band filter availability, we designed a custom multi-band filter that is now available from Chroma Technology (part number: ET525/50-800/200m).

Assuming comparable starting locations of the ectopic excitation, we compared conduction by assessing the timing of the V_m_-peak in two regions at different distances from the excitation point. No significant differences were found for electrically and mechanically induced ectopic beats, whether assessed in an equatorial (Fig. II) or axial direction (Fig. III) relative to the ventricular gross anatomy (Welch’s t test was used because of the possibility of unequal variances). In addition, propagation speeds (ranging from 0.64 to 0.66 mm × ms^-1^) were similar to those found in other whole-heart rat studies, paced electrically near the base.^3^ For the two rat hearts shown above, all ectopic beats were induced 170 ms after maximum QR upstroke slope.

**Online Figure IV** Circuit diagrams & parts list relevant to

optical mapping component of instrument (8 pages).


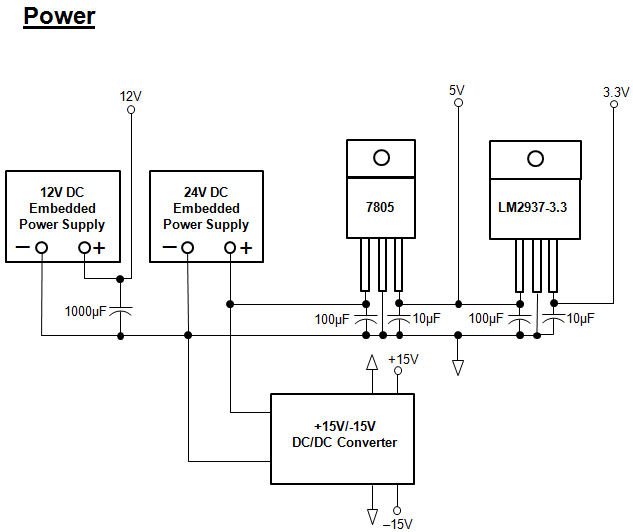


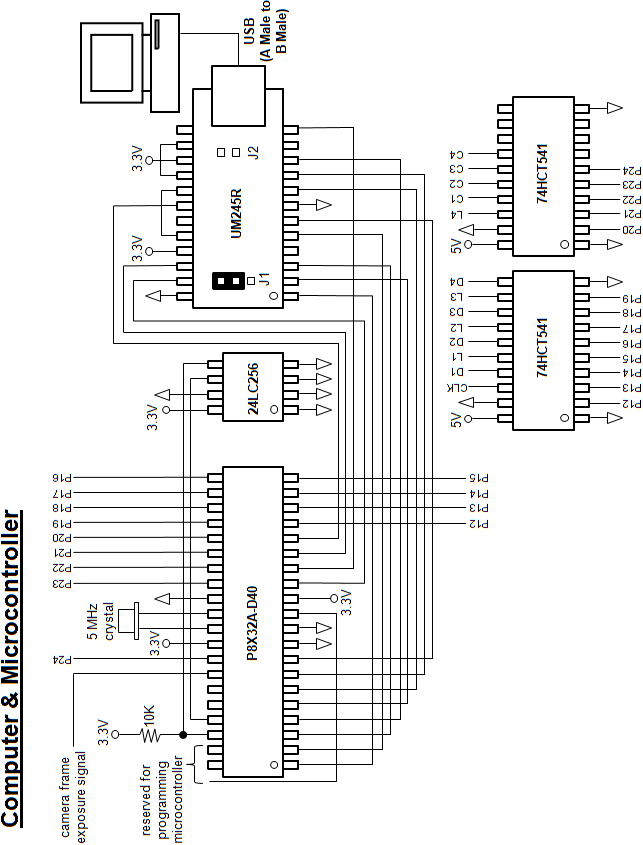


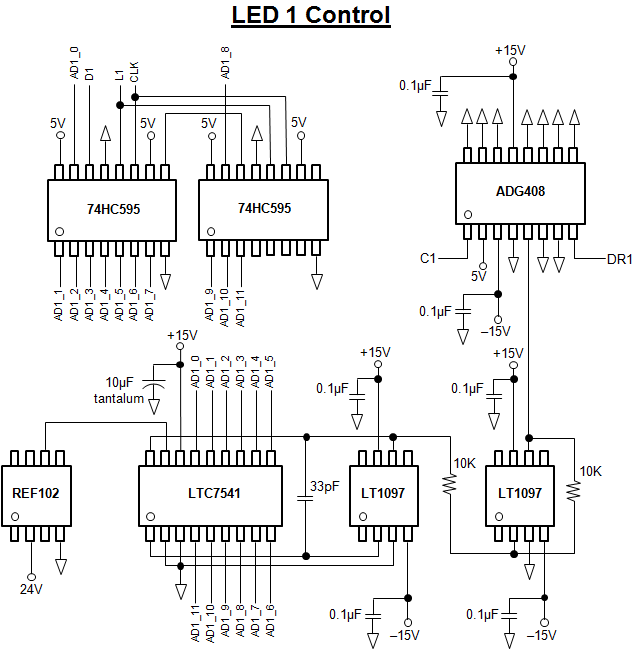


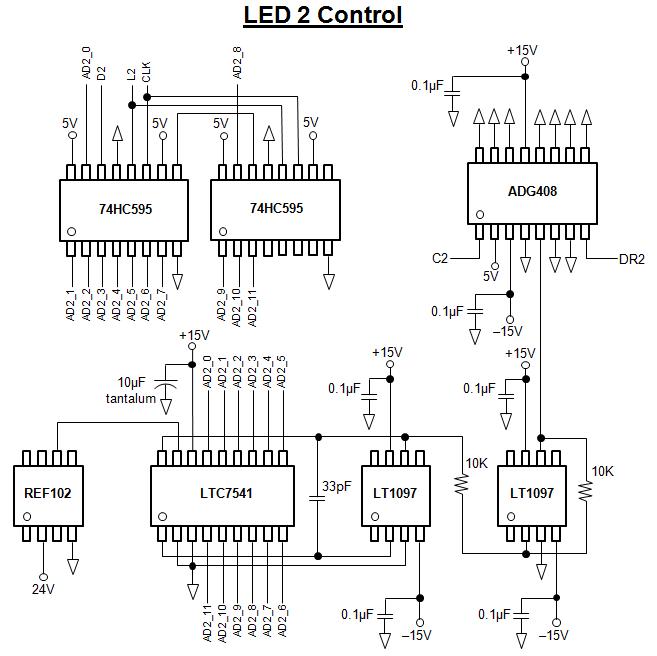


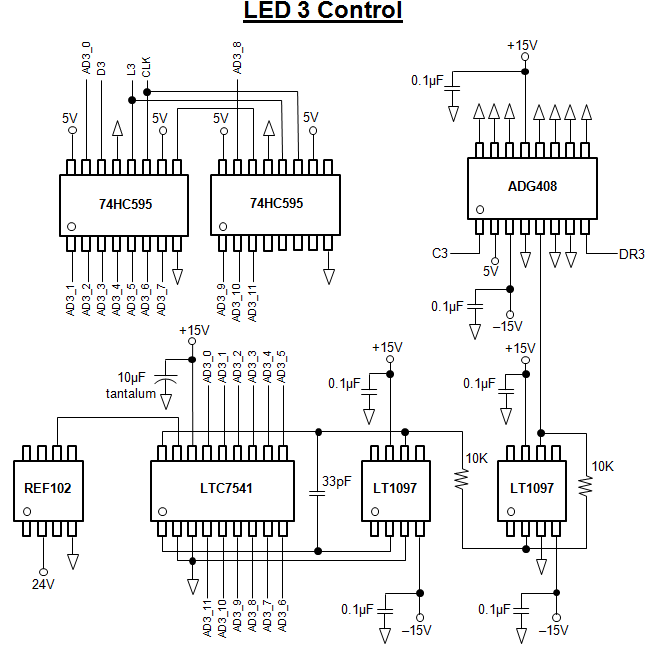


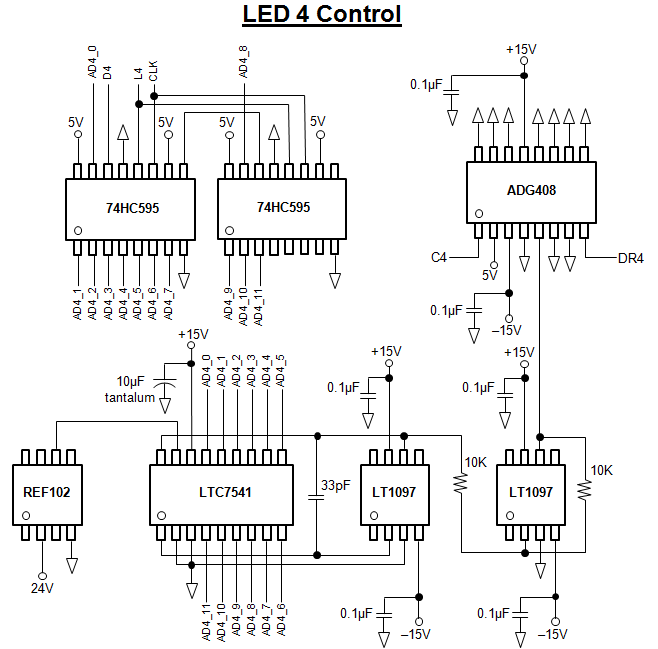


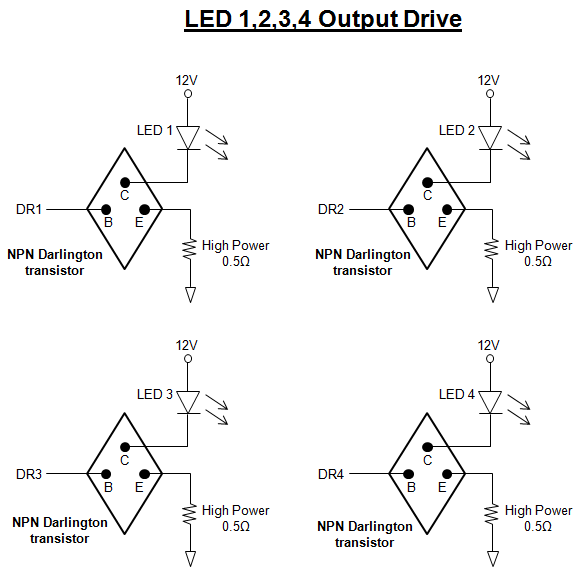


**Parts List (Key Components)**

**Name Part Number Manufacturer**

**Power:**

12V DC Embedded SP-150-12 RS Components

Power Supply

24V DC Embedded RS-15-24 RS Components

Power Supply

7805 LM340T-5.0 National Semiconductor

LM2937-3.3 LM2937ET-3.3 National Semiconductor

+15V/-15V VAWQ6-Q24-D15H CUI Inc

DC/DC Converter

**Computer & Microcontroller:**

P8X32A-D40 P8X32A-D40 Parallax Inc

5MHz crystal ECS-50-20-4 ECS Inc

24LC256 24LC256-I/P Microchip Technology

UM245R UM245R Future Technology Devices International

74HCT541 CD74HCT541E Texas Instruments

**LED 1 Control (same for LED 2 Control, LED 3 Control & LED 4 Control):**

74HC595 74HC595N NXP Semiconductors

ADG408 ADG408BNZ Analog Devices Inc

REF102 REF102AP Texas Instruments

LTC7541 LTC7541AKN Linear Technology

LT1097 LT1097CN8 Linear Technology

**LED 1,2,3,4 Output Drive:**

LED 1 CBT-90-B-C11 Luminus Devices

LED 2 CBT-90-R-C11 Luminus Devices

LED 3 UVMAX325-HL-15 Roithner Lasertechnik

LED 4 NCSU034A Nichia Corp

NPN Darlington MJ11016G ON Semiconductor

transistor

High Power 0.5Ω HS100 R5 J Arcol

**Online Figure V** Software for optical mapping component of instrument (7 Pages).

**Microcontroller Program**


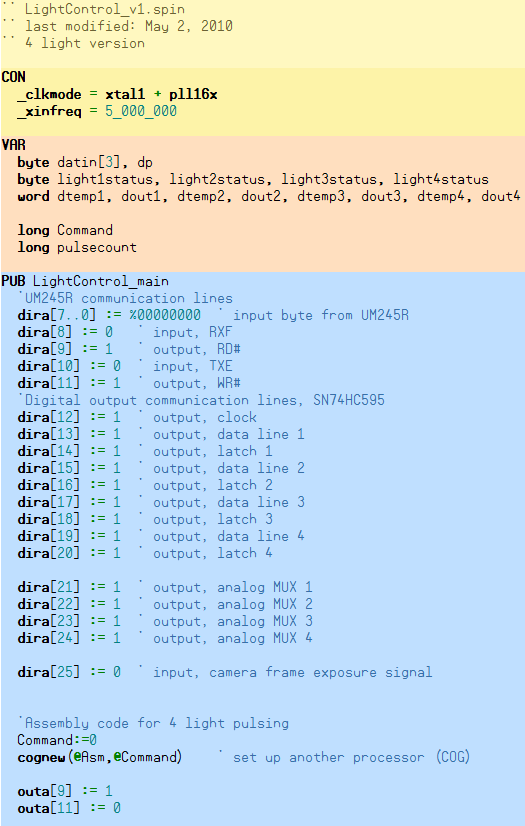


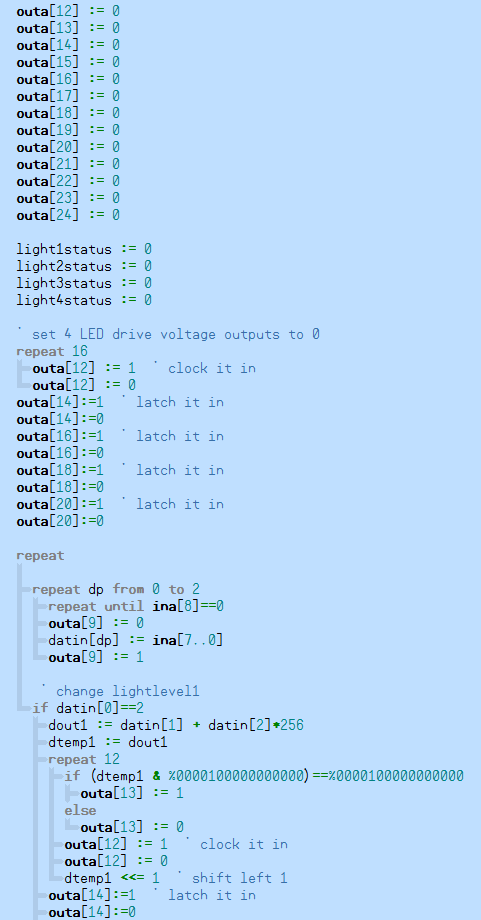


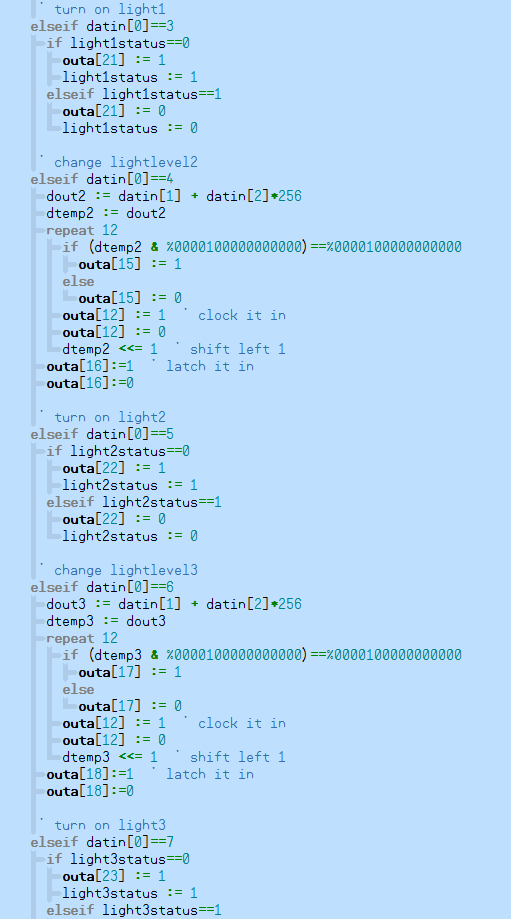


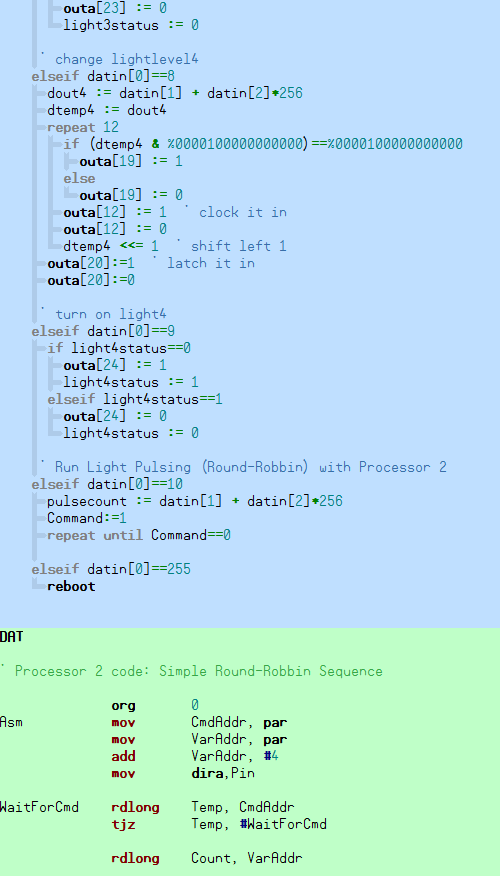


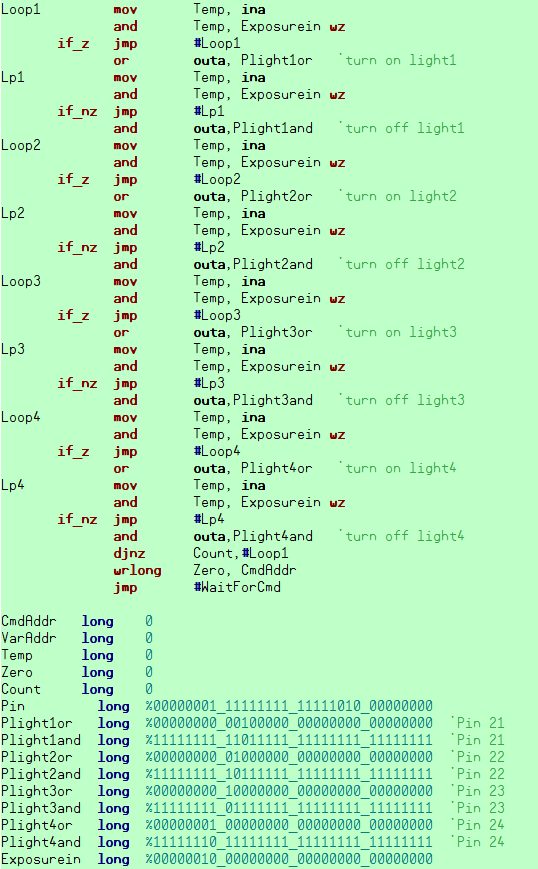


**MATLAB Commands to Communicate with Microcontroller**

**via UM245R**

**Setting up COM port:**

clear all;

s = serial('COM5');

set(s,'BaudRate',115200);

% clear buffer

c = get(s,'BytesAvailable');

if c~=0

fread(s,c);

end

fopen(s)

**Set LED 1 output level:**

volt = 2.50; % set DAC voltage output

num = round(4096*volt/10);

num2 = floor(num/256);

num1 = num - num2*256;

fwrite(s, [2 num1 num2], 'uint8');

**Turn on/off LED 1:**

fwrite(s, [3 0 0], 'uint8');

**Set LED 2 output level:**

volt = 2.50; % set DAC voltage output

num = round(4096*volt/10);

num2 = floor(num/256);

num1 = num - num2*256;

fwrite(s, [4 num1 num2], 'uint8');

**Turn on/off LED 2:**

fwrite(s, [5 0 0], 'uint8');

**Set LED 3 output level:**

volt = 1.50; % set DAC voltage output

num = round(4096*volt/10);

num2 = floor(num/256);

num1 = num - num2*256;

fwrite(s, [6 num1 num2], 'uint8');

**Turn on/off LED 3:**

fwrite(s, [7 0 0], 'uint8');

**Set LED 4 output level:**

volt = 1.50; % set DAC voltage output

num = round(4096*volt/10);

num2 = floor(num/256);

num1 = num - num2*256;

fwrite(s, [8 num1 num2], 'uint8');

**Turn on/off LED 4:**

fwrite(s, [9 0 0], 'uint8');

**Run round-robbin LED excitation sequence:**

num = 2500; % total number of camera frames

num = num/4; % adjust for 4 lights, round-robbin sequence

num2 = floor(num/256);

num1 = num - num2*256;

fwrite(s, [10 num1 num2], 'uint8');

**Supplemental References**

**1.** Scaduto RC, Jr., Grotyohann LW. Hydrolysis of Ca^2+^-sensitive fluorescent probes by perfused rat heart. Am J Physiol Heart Circ Physiol 2003;285:H2118-2124.

**2.** Matiukas A, Mitrea BG, Qin M, et al. Near-infrared voltage-sensitive fluorescent dyes optimized for optical mapping in blood-perfused myocardium. Heart Rhythm 2007;4:1441-1451.

**3.** Nygren A, Kondo C, Clark RB, Giles WR. Voltage-sensitive dye mapping in Langendorff-perfused rat hearts. Am J Physiol Heart Circ Physiol 2003;284:H892-902.
